# Antiviral Compounds for Blocking Arboviral Transmission in Mosquitoes

**DOI:** 10.3390/v13010108

**Published:** 2021-01-14

**Authors:** Shengzhang Dong, George Dimopoulos

**Affiliations:** W. Harry Feinstone Department of Molecular Microbiology and Immunology, Bloomberg School of Public Health, Johns Hopkins University, Baltimore, MD 21205, USA; sdong13@jhu.edu

**Keywords:** mosquito-borne viral diseases, antiviral drugs, small molecules, arboviral transmission cycle, dengue virus, Zika virus, *Aedes aegypti*

## Abstract

Mosquito-borne arthropod-borne viruses (arboviruses) such as the dengue virus (DENV), Zika virus (ZIKV), and chikungunya virus (CHIKV) are important human pathogens that are responsible for significant global morbidity and mortality. The recent emergence and re-emergence of mosquito-borne viral diseases (MBVDs) highlight the urgent need for safe and effective vaccines, therapeutics, and vector-control approaches to prevent MBVD outbreaks. In nature, arboviruses circulate between vertebrate hosts and arthropod vectors; therefore, disrupting the virus lifecycle in mosquitoes is a major approach for combating MBVDs. Several strategies were proposed to render mosquitoes that are refractory to arboviral infection, for example, those involving the generation of genetically modified mosquitoes or infection with the symbiotic bacterium *Wolbachia*. Due to the recent development of high-throughput screening methods, an increasing number of drugs with inhibitory effects on mosquito-borne arboviruses in mammalian cells were identified. These antivirals are useful resources that can impede the circulation of arboviruses between arthropods and humans by either rendering viruses more vulnerable in humans or suppressing viral infection by reducing the expression of host factors in mosquitoes. In this review, we summarize recent advances in small-molecule antiarboviral drugs in mammalian and mosquito cells, and discuss how to use these antivirals to block the transmission of MBVDs.

## 1. Introduction

Arthropod-borne viruses (arboviruses) are transmitted to humans or other vertebrates by arthropod vectors such as mosquitoes, ticks, and flies, and are mainly members of the *Flaviviridae*, *Togaviridae*, and *Bunyaviridae* families [1]. These viruses are transferred to a vertebrate host through saliva when an infected arthropod vector feeds on blood. There are more than 250 species of arboviruses, and at least 80 of them cause human diseases, including hemorrhagic fever, encephalitis, arthritis, and meningitis [2]. Diseases caused by arboviruses account for a major portion of vector-borne diseases (VBDs), and 80% of the global population lives in areas in which at least one VBD is endemic [3].

The recent emergence and re-emergence of mosquito-borne viral diseases (MBVDs) caused by, for example, the Zika virus (*Flaviviridae*, ZIKV), chikungunya virus (*Togaviridae*, CHIKV), dengue virus (*Flaviviridae*, DENV), Japanese encephalitis virus (*Flaviviridae*, JEV), West Nile virus (*Flaviviridae*, WNV), yellow fever virus (*Flaviviridae*, YFV) have raised international concerns, and continue to have a major impact on global public health and socioeconomic systems [4]. MBVDs are transmitted by culicine mosquitoes, mainly those in the *Aedes* and *Culex* genera. There are about 50 to 100 million infections by the four serotypes of DENV (DENV1 to DENV4) every year resulting in approximately 25,000 deaths [5]. CHIKV caused outbreaks in southern Europe in 2006–2007 and a small outbreak in the state of Florida, USA in 2014 [6,7]. The most recent ZIKV outbreak (2015–2016) in the Americas had a significant global effect on health and economic development [8]; during that outbreak, it was estimated that 1.5 million people had been infected in Brazil, with over 3500 cases of microcephaly reported. 

Current control methods for MBVDs are insufficient because there is a lack of effective vaccines and medications to control key MBVDs (dengue, Zika, and chikungunya). Thus, novel control strategies are urgently needed to supplement traditional vector-control methods that still represent the main responses to most mosquito-borne diseases in endemic areas. A few novel control approaches have recently been proposed for the fight against MBVDs, including gene-drive-based mosquito population suppression and modification, and the release of *Wolbachia*-infected mosquitoes to either suppress or render mosquito populations refractory to the viral pathogens [9]. In this review, we summarize another control strategy relying on small-molecule antiviral compounds to block arboviral transmission in mosquitoes. 

The arboviral transmission cycle in nature involves the circulation of the virus between vertebrate hosts and arthropod vectors. To establish a successful transmission cycle, an arbovirus needs to infect and replicate in cells of both vertebrates and arthropod vectors, and to reach a certain titer to render the mosquito infectious. One of the main approaches to impeding the transmission of MBVDs in nature is to suppress arboviral infection/replication in mosquitoes; this concept is supported by proof-of-principle studies using genetically modified mosquitoes that are refractory to arboviruses such as DENV and ZIKV [10,11,12,13,14]. Another novel approach is the use of antiviral compounds to inhibit arboviral infection in the vector [15].

In recent years, the rapid development of high-throughput screening methods has led to the discovery of a plethora of small-molecule compounds that can inhibit arboviral infection and/or replication in vertebrate cells [16,17,18,19,20,21,22], and a few also proved to be effective in mosquito cells. These anti-arboviral compounds can impede the circulation of arboviruses between arthropods and humans by either rendering arboviruses more vulnerable in humans or directly inhibiting viral infection and replication in mosquitoes. More importantly, most of these small-molecule drugs have already been approved by the Food and Drug Administration (FDA) or are currently evaluated in clinical trials. In this review, we mainly focus on current knowledge concerning the small-molecule compounds that can block the transmission of MBVDs between mosquitoes and humans, and the possible anti-arboviral mechanisms of these compounds in mosquitoes. 

## 2. Anti-Arboviral Compounds Suppressing Viral Infection in Mammalian Cells 

Small-molecule drugs are organic compounds of low molecular weight (usually under 900 Daltons) that regulate particular biochemical processes in ways that can result in the treatment or prevention of disease. Small-molecule drugs are attractive because they are easily synthesized due to their relatively low molecular weight and simple chemical structure, and they are generally highly stable [23]. By means of cell-based high-throughput screening, hundreds of small-molecule drugs were identified that can inhibit at least one type of arbovirus in mammalian cells [16,17,18,24,25,26]. In general, these screenings were mainly concentrated on DENV, and recently on ZIKV. These anti-arboviral drugs were derived from a diversity of sources, including plant products, bacterial metabolites, and chemically synthesized compounds. Some of the drugs exhibited a broad spectrum of anti-arboviral activity in various mammalian cells in culture (Table 1) and in mice in vivo, but the antiviral nature of these drugs still needs to be further explored in mosquito biology. For example, nanchangmycin, a natural product of *Streptomyces nanchangensis*, can inhibit the production of several viruses in the *Flaviviridae* (DENV2, ZIKV, and WNV) and *Togaviridae* (SINV, CHIKV) families [27]. 

The modes of action of anti-arboviral drugs in mammalian cells are diverse, but they mainly target viral proteins and/or mammalian cell proteins to inhibit viral production, RNA synthesis, or entry, attachment, and secretion (Table 1). For example, sofosbuvir, an FDA-approved nucleotide polymerase inhibitor for the hepatitis C virus, inhibited the RNA polymerase from various *Flaviviridae* family viruses, including ZIKV, DENV, and YFV [28,29,30,31], and interacted with CHIKV NsP4 to repress viral production [32]; suramin, an approved antiparasitic drug, blocked the replication of CHIKV by inhibiting an earlier post-attachment step in the CHIKV replicative cycle in vitro and viral RNA synthesis in vivo [33], suppressed ZIKV replication by interfering with the attachment and release of infectious progeny from host cells [34], and inhibited DENV production by interfering with attachment to host cells [35]. The diversified antiviral mechanisms of these drugs make it more difficult to develop drug resistance in host cells. This assertion was recently supported by a study showing that JG40 (an HSP70 inhibitor) had antiviral activity against a number of flaviviruses, including DENV2, WNV, and YFV, retaining a comparable inhibitory effect on DENV2 infection in mammalian cells (Huh-7) that were continuously treated for 10 passages [36]. 

Some antiviral drugs have a conserved mode of action on a number of different arboviruses in host cells. For example, niclosamide, an FDA-approved antiparasitic drug, inhibited the entry and transmission of DENV, CHIKV, and perhaps other viruses such as ZIKV by hindering endosomal acidification and interfering with pH-dependent membrane fusion [37,38,39,40]. Drugs that exerted conserved antiviral mechanisms against multiple arboviruses in different mammalian cell types suggested that they may also have had a similar inhibitory effect in mosquito cells. This hypothesis is supported by recent studies that showed that 4-hydroxyphenyl retinamide (4-HPR) and mycophenolic acid (MPA), which inhibit pan-*Flaviviridae* arboviral infection in mammalian cells [41,42,43,44] also had a similar inhibitory effect on DENV2 and ZIKV infection in mosquito cells (C6/36) and mosquito (*Ae. aegypti*) midguts [15,45]. 

## 3. Antiarboviral Compounds Suppressing Virus Infection in Mosquito Cells

As compared to mammalian cells, only a few small-molecule drugs were tested in mosquitoes or mosquito cells, and most of them represented already confirmed antiarbovirals in mammalian cells. To the best of our knowledge, there is no published high-throughput screening of small-molecule compounds for anti-arboviral activity in mosquito cells, while there is on *Drosophila melanogaster* cells [70,71]. 

The arboviral infection cycle in mosquito cells, like that in mammalian cells, involves an initial interaction between viral surface proteins and receptor molecules on the host cell surface, followed by receptor-mediated endocytosis to internalize the viral particles within the cytoplasm, viral assembly in the endoplasmic reticulum (ER), and lastly secretion of produced mature virions [72,73,74]. The arboviral infection cycle is the main target of small-molecule compounds to suppress viral production in mosquito cells (Table 2). For example, chlorpromazine, monodanslycadervine, and dynasore are predicted inhibitors of receptor-mediated endocytosis that block clathrin-mediated endocytosis, which is part of viral entry into mosquito cells [75,76,77]. In fact, this is believed to be the main viral entry pathway for *Flaviviridae* arboviruses in mosquito cells [74]. In addition, an acidic pH compartment is required for viral infection of mosquito cells; therefore, small-molecule compounds that affect pH levels could inhibit viral production. This conjecture was supported when the production of DENV1-2 and WNV in C6/36 cells was shown to be inhibited by the addition of ammonium chloride to the cell medium during viral infection [72,76,77], and further studies revealed that ammonium chloride repressed viral replication by raising the pH, thereby blocking the acidification of the endolysosomal network and the fusion of the viral envelope with endosomal membranes.

Although mammalian and mosquito cells responded differently upon viral infection, some antiviral drugs employed a similar mode of action in both types of cells to inhibit arbovirus infection. For example, benzodiazepine inhibited YFV by targeting the viral NS4B protein in both human (HEK293 and Huh-7) and mosquito (C6/36) cells [82], and showed similar resistance profiles in NS4B mutants in these three cell types; 2’C-methyladenosine and JG18 significantly reduced DENV2 production by suppressing viral RNA synthesis and viral protein expression in both mammalian (Huh-7) and mosquito (C6/36) cells [36]. However, most antiviral drugs have different modes of action with regard to arboviral infection in mammalian and mosquito cells. For instance, mycophenolic acid (MPA) inhibited the synthesis and accumulation of viral RNA (DENV2, YFV, and WNV) in various mammalian cells [42], whereas it suppressed DENV2 infection in *Ae. aegypti* midguts by reducing the expression of inosine-5′-monophosphate dehydrogenase (IMPDH) [15]. These distinct modes of action led to differences in the inhibitory activity of particular antiviral drugs in mammalian and mosquito cells. For instance, sulfated polysaccharides, including heparin, glucans, and carrageenans, were shown to be potent and selective inhibitors of arboviral infection in various mammalian cells [85,86,87,88], but they did not possess inhibitory effects in mosquito cells [89,90], likely because of a lack of adequate heparan sulfate (HS) in C6/36 cells; however, one particular type of sulfated polysaccharide, iota-carrageenan, did exhibit a moderate inhibitory effect on DENV2 infection in mosquito cells, although inhibition is weak when compared to that in mammalian cells [84]. This effect resulted from the antiviral activity that iota-carrageenan exerted during the initiation of replication in mammalian cells, thereby affecting DENV2 virion binding; in contrast, inhibitory activity in mosquito cells likely proceeded through the induction of cell proliferation and a reduction in protein synthesis [84]. 

## 4. Anti-Arboviral Compounds with Mosquitocidal Activity

Some anti-arboviral drugs possess both mosquitocidal and anti-arboviral activities in mosquito cells. For instance, ivermectin, a broad-spectrum antiparasitic agent, was demonstrated to have a toxic effect in C6/36 cells, and mosquitocidal activity in *Aedes* and *Anopheles* mosquitoes [45,91,92]. Ivermectin effectively inhibited infection with DENV2, JEV, YFV, and CHIKV in mammalian cells [50,55], but did not inhibit ZIKV in either mosquito cells or midguts when the concentration used for the assay was lower than the toxic level for cells or adult females was [45]. Cyclosporin, a broad-spectrum anti-arboviral (effective against DENV1-2, YFV, VSV, and ZIKA) [18,53], did not inhibit ZIKV in C6/36 cells but exhibited strong larvicidal activity against *Culex pipiens autogenicus* [93], adulticidal activity against *Ae. aegypti,* and toxicity in C6/36 cells [45]. When compared to conventional insecticides, these anti-arboviral compounds were safe in humans, and mosquitoes have not developed resistance to any of them. Thus, they have great potential for the development of therapeutics that may also kill mosquitoes when they blood-feed. 

## 5. Anti-Arboviral Drug Mechanisms in Mosquitoes

When a mosquito ingests a viremic blood meal from an infected host, arboviruses are transferred to the mosquito midgut lumen, from where they infect epithelial cells; after replication, they are disseminated into the hemocoel. The viruses then infect and replicate in the salivary glands, and are released into the saliva, from which they can be transmitted to another host through a mosquito bite [94]. Thus, disrupting the arbovirus infection cycle in mosquitoes may be achieved by inhibiting viral infection/replication in midgut epithelial cells and salivary glands by either altering midgut microbiota or boosting the mosquitoes’ antiviral immunity, targeting viral RNA with a small interfering RNA (siRNA) pathway, or functionally disrupting viral host factors. Theoretically, small-molecule antiviral drugs could block the virus by interfering with any factors that are essential for viral infection/replication. 

Small molecules that could enhance the mosquitoes’ antiviral defense represent candidate anti-arboviral drugs. The systemic defense against arboviral infection in mosquitoes involves multiple tissue types, including the midgut, hemocytes, the central nervous system, and salivary glands [95,96], and the innate immune response of mosquitoes is a key determinant of the successful transmission of arboviruses. Numerous studies showed that managing mosquitoes’ innate immune pathways, such as the siRNA pathway [97,98], the Janus kinase-signal transducer and activator of transcription (JAK/STAT) pathway [10,99], and the Toll pathway [100], can reduce viral production and infection prevalence in *Ae. aegypti* mosquitoes. In addition to innate immunity, apoptosis also restricted arbovirus infection in mosquitoes, and induced apoptosis was shown to effectively reduce midgut infection, replication, and dissemination by SINV in *Ae. aegypti* [101]; furthermore, apoptosis limits WNV infection of midgut epithelial cells and the dissemination of virions from *Culex pipiens pipiens* midguts [102]. The retinoic acid (RA) derivative 4-HPR and a potential cancer-preventive agent that acts by inducing apoptosis in cancer cells, can inhibit ZIKV and DENV infection in mammalian cells [41,44] and mosquito midguts [45], and this inhibition likely occurs through an influence on apoptosis in the mosquitoes.

Small-molecule compounds that interfere with viral host factor (HFs) function have the potential to suppress viral infection or replication. HFs are mosquito factors that are essential for the infection and replication of an arbovirus in mosquito cells [95], and numerous HFs were recently identified in mosquitoes. For example, prohibitin was shown to be a DENV HF that can conceivably act as a receptor protein to mediate DENV entry into mosquito cells [103]; a saliva-specific protein, *Ae. aegypti* venom allergen-1 (AaVA-1), promotes DENV and ZIKV transmission in mammalian and mosquito cells [104]; and an *Ae. aegypti* C-type lectin, mosGCTL-1, interacts with WNV and facilitates infection in *Ae. aegypti* and *Culex quinquefasciatus* [105]. Thus, any small-molecule drugs that interfere with the expression or function of HFs in mosquitoes could hamper arboviral transmission between mosquitoes and humans. Moreover, some HFs are conserved between humans and mosquitoes. For example, vATPase plays a fundamental role in viral membrane fusion as a vacuolar proton pump that acidifies vacuoles [106], and the inhibition of vATPases with bafilomycin (BAF) was shown to suppress SINV and ZIKV infection in mammalian cells [107,108] and JEV and DENV2 in mosquito cells, as well as DENV2 in *Ae. aegypti* midguts [15,76]. 

## 6. Application of Antiviral Compounds in Mosquitoes

To block arboviruses in mosquitoes, antiviral drugs must be delivered to the adult stage, ideally in a way by which they could become exposed to the midgut tissue, which is the entry point and key replication site for arboviruses. Thus, these drugs could be implemented as part of attractive toxic sugar baits (ATSBs), a novel control approach that was recently employed for adult mosquito control in the field [109]. The concept exploited the sugar-feeding behavior of mosquitoes, inducing them to feed on artificial nectar containing an insecticidal ingredient. ATSBs could reduce mosquito populations and thus the probability of arboviral transmission; however, ATSBs also attracted and killed nontargeted beneficial insects such as honeybees and parasitoid wasps [110]. If insecticides were replaced with small-molecule antiviral drugs, the baits would be rendered more environmentally friendly, and the accidental killing of beneficial insects would be reduced. Thus, antiviral drugs could be considered as an alternative ingredient in ATSBs. 

Insecticide-treated bed nets (ITNs) are effective in preventing VBDs, and they achieved great success in preventing malaria, which is mainly transmitted by *Anopheles* mosquitoes [111,112]. ITNs were also proved effective in controlling *Aedes* mosquitoes and reducing dengue virus prevalence [113,114]; however, resistance to widely used insecticides such as pyrethroids, organochlorines, and organophosphates developed in mosquito populations, rendering ITNs less effective for controlling VBDs. A recent study showed that using specific antimalarial drugs instead of insecticides can achieve similar transmission-blocking effects on *Plasmodium* in *Anopheles* mosquitoes [115]. Thus, the application of antiviral drugs to bed nets can be reasonably considered for blocking arboviral transmission in mosquitoes. For the safety of the environment and humans, small antiviral drugs could also be applied with indoor residual spraying (IRS) to prevent arboviral transmission.

## 7. Future Prospects and Challenges

The advantages of using small-molecule drugs to impede arboviral transmission are obvious: they are safe for both humans and the environment, and they can be transferred from humans to mosquitoes when patients receive treatment with the drugs; however, because of significant differences in drug metabolism and viral pathogenesis between vertebrates and insects, and the fact that mosquitoes lack adaptive immune responses and are dependent on innate immunity for defense against viral infection, many small-molecule anti-arboviral drugs that work in mammalian cells do not have a similarly inhibitory effect on arboviral infection/replication in mosquitoes and mosquito cells. It is challenging to identify anti-arboviral drugs that are effective in mosquitoes on the basis of the current pool of anti-arboviral drugs in mammalian cells. In addition, most small-molecule drugs are costly. Nevertheless, a few antiviral drugs have an inhibitory effect on arboviral infection in both mosquitoes and mosquito cells, demonstrating that it is possible to use antiviral compounds to stop the spread of VBDs in mosquitoes. Studies should focus on identifying small-molecule drugs with strong transmission-blocking activity against key arboviruses in mosquitoes. These drugs have advantages for therapeutic activity in mammals and transmission-blocking activity in mosquitos; thus, such dual-action drugs could limit the transmission of arboviruses from infected people who are being treated.

## Figures and Tables

**Table 1:** Small-molecule compounds with a broad spectrum of antiarboviral activity in mammalian cells.

Small-Molecule Compound	Description	Viruses	Cells/Hosts	Mode of Action	References
4-Hydroxyphenyl retinamide	Vitamin A acid analog with anti-proliferative activity	DENV1-4, WNV, ZIKV	HEK293T, Vero, Huh-7	Inhibits steady-state accumulation of viral genomic RNA	[41,43,44]
6-Azauridine	Synthetic triazine analog	DENV1-2, DENV4, ZIKV, JEV, YFV	Vero	Inhibits viral multiplication	[46,47]
6-Deoxyglucose-diphyllin	Naphthalene-derived bioactive phytoconstituent molecule	DENV1, WNV, JEV, ZIKV	Vero, HT1080, CHME3, C57BL/6 Ifnar1^−/−^ mice	Targets cellular endosomal acidification, preventing viral entry	[48]
AP30451	Secondary sulfonamide compound	DENV2, WNV, YFV	Huh-7, BHK21	Inhibits translation of flaviviral RNA	[49]
Candesartan cilexetil	Angiotensin II receptor inhibitor	DENV2, ZIKV, CHIKV, KUNV	JEG-3, HEK293T, HeLa	Targets postentry stage(s) of ZIKV replication cycle	[24]
Berberine	Relatively nontoxic isoquinoline alkaloid	CHIKV, SINV, SFV	BHK21, Huh7.5, C57BL6/J WT mice	Reduces CHIKV-induced MAPK signaling	[50,51]
Castanospermine	Alphaglucosidase inhibitor	DENV1-4, YFV, WNV	Huh-7, BHK-21	Inhibits dengue virus secretion and infectivity of viral particles	[52]
Cyclosporin	Immunosuppressive agent	DENV1-2, YFV, VSV, ZIKA	Huh-7, Vero	Suppresses viral RNA synthesis	[18,53]
Glycyrrhizin	Natural triterpene glycoside	DENV1-2, DENV4, YFV, ZIKV, JEV	Vero	ND	[46,54]
Ivermectin	Antimalarial drug	DENV2, JEV, YFV, CHIKV	Vero, BHK-21, Huh-7	Targets NS3 helicase activity	[50,55]
JG40	Hsp70 inhibitor	DENV2, DENV4, KUNV, YFV, JEV	MDDC, Huh-7	Inhibits replication of viral RNA and packaging of viral particles	[36]
Lanatoside C	FDA-approved cardiac glycoside	DENV1-4, CHIKV, SINV	HuH-7, U937, HUVEC, BHK-21	Inhibits DENV-2 viral protein and viral RNA synthesis	[56]
Mycophenolic acid	Inhibitor of inosine monophosphate dehydrogenase	DENV2, YFV, WNV	Hep3B, HepG2	Prevents synthesis and accumulation of viral RNA	[42]
Nanchangmycin	Natural product of *Streptomyces nanchangensis*	DENV2, ZIKV, WNV, SINV, CHIKV	U2OS, HUVECs, UtMECs	Blocks viral entry into cells	[27]
Niclosamide	Antiparasitic drug	DENV2, CHIKV, SINV, SFV, ZIKV	BHK-21, U2OS, A549, Neuro-2a	Limits viral entry, inhibits viral release, and hinders endosomal acidification	[38,39,40]
Nitazoxanide	Antiparasite infection	DENV2, CHIKV, SINV, SFV, JEV, YFV	BHK-21, U2OS	Inhibits viral maturation and early–mid stage of JEV infection	[39,57]
NITD008	Adenosine nucleoside analog	DENV1-4, YFV, WNV, ZIKV	BHK-21, A549, and Huh-7, Vero	Inhibits RNA-dependent RNA polymerase activity	[58,59]
NITD203	Adenosine nucleoside analog	DENV1-4, YFV, WNV	A549	Inhibits flaviviral RNA synthesis	[60]
Orlistat (tetrahydrolipstatin)	FDA-approved drug used as weight-loss medication	DENV1-4, ZIKV, CHIKV, JEV	HepG2, HEK293T	Inhibits correct formation of DENV viral particles and release to the cells	[61,62]
Posaconazole	Potent antifungal drugs	DENV1-2, DENV4, ZIKV, YFV	Vero, BHK-21	Inhibits RNA replication stage	[63]
Quinestrol/raloxifene	Estrogen receptor modulators	DENV2, ZIKV, WNV (KUNV)	Huh-7, HTR8/SVneo	Inhibit viral RNA replication	[21]
Ribavirin (RBV)	Broad-spectrum antiviral drug	DENV1-4, CHIKV, WNV, YFV	LLC-MK2, Hep3B, HepG2, Vero	Interferes with synthesis of DENV mRNA	[42,64,65,66,67]
Sofosbuvir	Antihepatitis C virus drug	DENV2, ZIKV, CHIKV, YFV	HuH-7, HepG2, Vero, C57BL/6 mice, A129-/- mice	Inhibits DENV NS5 polymerase activity, targets CHIKV NsP4, and reduces viral genome replication and infectious viral particle production	[28,29,30,31,32]
Suramin	Approved antiparasitic drug	DENV2, CHIKV, SINV, SFV, ZIKV	CHO-K1, Vero, BHK-21	Inhibits CHIKV RNA synthesis, and interferes with ZIKV attachment and release	[33,34,35]
Tomatidine	Natural steroidal alkaloid	DENV1-4, ZIKV, CHIKV	A549, Huh-7	Postentry step of viral replication cycle	[68,69]
Valinomycin	Potassium-specific transporter	RVFV, LACV, ZIKV	Huh-7, Vero	Precludes viral replication by altering cellular potassium ions	[22]

Abbreviations: DENV1-4: dengue virus serotype 1-4; ZIKV: Zika virus; CHIKV: chikungunya virus; WNV: West Nile virus; KUNV: Kunjin virus; YFV: yellow fever virus; SINV: Sindbis virus; JEV: Japanese encephalitis virus; SFV: Semliki Forest virus; RVFV: Rift Valley fever virus; LACV: La Crosse virus; VSV: vesicular stomatitis virus. ND: not determined.

**Table 2:** Small-molecule compounds with anti-arboviral activity in mosquitoes and mosquito cells.

Small-Molecule Compound	Description	Viruses	Cells/Hosts	Mode of Action	References
1E7-03	Tetrahydroquinoline derivative	RFV	C6/36	Blocks viral RNA transcript/protein interactions to inhibit viral RNA production	[78]
2’C-methyladenosine	NS5 polymerase inhibitor	DENV2	C6/36	Suppresses viral RNA synthesis and viral protein expression	[36]
4-Hydroxyphenyl retinamide	Vitamin A acid analog with antiproliferative activity	DENV2, ZIKV	C6/36, *Ae. aegypti*	Targets ZIKV nonstructural protein 5	[44,45]
5-Fluorouracil	Potent antitumor agent; affects pyrimidine synthesis.	DENV1, ZIKV	C6/36	Affects RNA incorporation into DENV1 virions	[79,80]
Ammonium chloride	Systemic and urinary acidifying salt	DENV1-2, WNV	C6/36	Influences fusion of viral envelope with endosomal membrane	[75,76,77]
Bafilomycin	Macrolide antibiotic; inhibitor of vacuolar H^+^-ATPase	DENV2, JEV	C6/36, *Ae. aegypti*	Inhibits synthesis of mosquito vATPase	[15,81]
Benzodiazepine	Common type of antianxiety drug	YFV	C6/36	Inhibits postentry step of YFV replication	[82]
Bisindolymaleimide	Highly selective protein kinase C (PKC)	WNV	C6/36	Inhibits trafficking of internalized WNV along the endosomal pathway	[75]
Bortezomib	Dipeptide boronic acid derivative and proteasome inhibitor	ZIKV	C6/36, *Ae. aegypti*	Affects ZIKV entry process	[45,83]
Chlorpromazine	D2 dopamine receptor and H1 histamine receptor antagonist	DENV1-4, WNV	C6/36	Blocks viral entry by inhibition of clathrin-mediated endocytosis	[75,76,77]
Clotrimazole	Specific inhibitor of Ca2+-activated K+ channels. It is an antifungal azole	ZIKV	*Ae. aegypti*	ND	[45]
Dansylcadaverine	Lysosomotropic agent	DENV1-2, WNV	C6/36	Inhibits clathrin-mediated endocytosis	[75,76,77]
Dynasore	Dynamin inhibitor	DENV2-4	C6/36	Blocks clathrin-mediated endocytosis	[76]
Genistein	Multiple tyrosine kinase inhibitor	WNV	C6/36	Inhibits phosphorylation of focal adhesion kinase	[75]
Imatinib mesylate	Known inhibitor of the c-Kit, Bcr-Abl, and PDGFR tyrosine kinases	ZIKV	*Ae. aegypti*	ND	[45]
Iota-carrageenan	Linear sulfated polysaccharide	DENV-2	C6/36	Induces cell proliferation and reduces protein synthesis	[84]
MKT077/JG18/JG40	Hsp70 inhibitors	DENV-2	C6/36	Inhibit viral RNA production	[36]
Mycophenolic acid	Non-nucleoside inhibitor of inosine monophosphate dehydrogenase	DENV2, ZIKV	C6/36, *Ae. aegypti*	Inhibits expression of Inosine-5′-monophosphate dehydrogenase in mosquitoes	[15,45,72]
U18666A	Intracellular cholesterol transport inhibitor	ZIKV	*Ae. aegypti*	ND	[45]

## Data Availability

Not applicable.
